# Cochlear Dysfunction in Chronic Otitis Media and Its Determinants

**DOI:** 10.22038/ijorl.2019.35045.2158

**Published:** 2020-03

**Authors:** Vijayalakshmi Subramaniam, Ali Ashkar, Sheetal Rai

**Affiliations:** 1 *Department of Otorhinolaryngology, Yenepoya Medical College, Mangalore city, Karnataka State, India.*; 2 *Assistant Surgeon, Government Taluk Hospital, Trikaripur Kasargod District, Kerala state, India.*

**Keywords:** Cochlear, Chronic otitis media, Sensorineural hearing loss

## Abstract

**Introduction:**

Long-standing chronic otitis media (COM) may lead to sensorineural hearing loss (SNHL). The present study aimed to evaluate the factors affecting the sensorineural component to counsel patients regarding the risk of SNHL at the event of untreated COM.

**Material and Methods:**

A time-bound cross-sectional study was conducted in the Department of Otorhinolaryngology at a tertiary care hospital. The study population included the study group comprising 137 patients with chronic suppurative otitis media (CSOM) and the control group which consisted of 137 individuals with the same age range and gender as the case study group. Moreover, the hearing was assessed using a pure tone audiogram and special tests of hearing.

**Results:**

Based on the findings of the present study, the SNHL was found in 71.4% of CSOM cases with an ear discharge duration of more than 5 years. The SNHL occurred in 55.2% and 44.7% of the cases with pars flaccida and of pars tensa perforations, respectively. In the case of pars tensa perforation, greater perforation size resulted in a steady increase in the odds of developing SNHL. Patients with subtotal and total perforations were at higher risk of developing SNHL.

**Conclusion:**

Patients with longer duration of disease, squamous type of disease, and larger size of pars tensa perforation had greater susceptibility to develop SNHL. Therefore, eradication of the disease from the middle ear and early reconstruction of the hearing mechanism during the course of the disease result in reducing the burden of SNHL.

## Introduction

Chronic suppurative otitis media (CSOM) is defined as a chronic inflammatory process that involves the middle ear cleft with recurrent episodes of ear discharge through a perforation in the tympanic membrane. It follows an episode of acute otitis media or may develop as a sequel of secretory otitis media (1). Hearing loss in CSOM can result from perforation of the tympanic membrane, disruption of ossicular chain (conductive hearing loss), outer hair cell damage caused by the diffusion of bacterial toxins into the inner ear (sensorineural hearing loss), or both (mixed hearing loss) (2,3). 

Previous studies have reported that inner ear is involved in middle ear inflammatory disease leading to sensorineural hearing loss (SNHL); therefore, proper treatments and follow-ups are recommended. The present study aimed to investigate the occurrence of SNHL in CSOM and find the correlation between SNHL and various factors, such as type and duration of the disease.

## Materials and Methods

A prospective observational cross-sectional study was carried out in the ear, nose, and throat diseases (ENT) department of a tertiary care hospital over a period of 18 months starting from October 2013. Clearance was obtained from the University's Ethics Committee. Patients who visited the ENT out-patient department with predominant signs and symptoms of CSOM and who had already been on medical treatment for the same were entered into the study. 

An equal number of volunteers of the same age and gender as the study group were enrolled as the control group. A detailed history was taken and a clinical examination was performed. Investigations, including complete blood count, erythrocyte sedimentation rate, and random blood sugar were conducted depending on the history and clinical examination. 

The sample size consisted of 274 individuals who were equally assigned to the study and control groups. Informed consent was obtained from all participants. Convenience sampling was employed for the selection of the participants.

**Study** **group selection**

The study group included individuals of both sexes between 11 to 45 years of age, with predominant symptoms and signs of CSOM. Pregnant women, patients aged less than 11 years, those with previous tympanomastoid surgery, those on ototoxic medication, those with history of head injury, those with history of meningitis or syphilis, those suffering from systemic diseases that may affect normal hearing (like Diabetes Mellitus), uncooperative patients, those with Hereditary/Familial hearing loss, post traumatic perforation of tympanic membrane and those with history of occupational noise exposure were excluded from the study.

**Control group selection**

A total of 137 volunteers with the same age and gender as the study group, who showed no signs and symptoms of chronic otitis media (COM) and did not undergo medical/surgical treatment for the same disease entered into the control group. Those with conditions mentioned in the exclusion criteria for the study group were excluded.

All the enrolled subjects underwent the following investigations:

**Pure-tone** **audiometry, namely high-frequency** **audiometry **

Pure tone audiometry was performed in an anechoic chamber with the help of the calibrated Interacoustics Clinical audiometer-AC-40 (Denmark). The obtained thresholds were used to quantitatively assess the degree of hearing loss using Clark’s (1981) modification of Goodman classification of severity of hearing loss (1965) (4). Additional to the testing across the conventional frequencies (250-8,000 Hz), each subject underwent high-frequency testing at frequencies ranging from 8,000-16,000 Hz. The transducer used for the testing was Koss R/80 supra-aural headphone.

**Short** **increment** **sensitivity** **index** **test**

At a sound level of 20 dB, a carrier tone was introduced into the patient's ear. A short increment of 5 dB was introduced after every 5 sec. There was a lapse of 5 sec between the increments with 50 msec of on-off time for the signal. The patient had to indicate hearing a brief increase in the loudness of the tone. 

Five such jumps were initially given to condition the patient. With the reduction in the increment size to 1dB, the short increment sensitivity index (SISI) test started. Around 20 such 1 dB increments were given and the subjects had to indicate each time they heard the increment. If 5 or more increments were heard consecutively, then such increments would be deleted. This would ensure that the response was to the change in intensity not to the time interval. If the subject did not respond to many consecutive increments, the size of the increment would be raised followed by a proper hearing test in order to retrain the patient. Consequently, false negative and false positive results were avoided.

**Short** **increment** **sensitivity** **index** **test variants:**

There are five variants as follows:

Classical SISI-A high score of 1 dB increments at 20dB sound level- is suggestive of a cochlear lesion. 

A low score of 2 to 5 dB increments at 20 dB sound level -is suggestive of a retrocochlear lesion.

A low score of 1 dB increments at high sound levels 75dB-is suggestive of a retrocochlear lesion. 

A lower score in one ear than the other with their thresholds being almost equal, when increment size varied from 1-5 dB at 20 dB sound level-is suggestive of a central lesion opposite the ear with the lower score. The difference in the rate at which scores increase when 1 dB increments are made at sound levels ranging from 20 dB to high levels (about 75dB) in 10 dB steps for both ears – is suggestive of a retrocochlear lesion. The location of the lesion would be on the same side as the ear which did not show normal increases in intensity (5).

## Results

A total of 274 participants entered into the current study. About 50% (n=137) of which were in the study group and the remaining 50% (n=137) in the control group. 

Out of 274 subjects, 46% (n=126) were female and 56% (n=148) were male. A total of 194 ears were included in the study group. Of these 59.2% (n=115) ears developed COM mucosal disease and 40.7% (n=79) ears had COM squamosal disease. 

Among the study group, conductive hearing loss (CHL) was observed in 47.3% of the subjects and mixed hearing loss was detected in 52.4%. While in the control group, the CHL and SNHL were observed in 5% and 5.1% of the subjects, respectively (Table.1).

**Table 1:** Hearing loss in patients with chronic otitis media and control group

	**COM**		**CONTROL**
Conductive hearing loss	Mild	42 (21.6%)	6 (3.0%)
	Moderate	50 (25.7%)	4 (2.0%)
Mixed hearing loss	Mild	51 (26.2%)	------------
	Moderate	51 (26.2%)	------------
SNHL	--------------	-------------	10 (5.1%)
Normal hearing	--------------	-------------	174 (89.6%)

COM: Chronic otitis media,SNHL:Sensorineural hearing loss

Mixed hearing loss was detected in 70% of patients with disease duration ranging within 4-5 years. This shows that with longer duration of the disease, the incidence of the mixed hearing loss increases (Table.2).

**Table 2:** Correlation of the duration of the disease and hearing loss

**Duration of disease**	**Hearing loss**			
	**Conductive hearing loss**		**Mixed hearing loss**	
	**Mild**	**Moderate**	**Mild**	**Moderate**
<1 year 25.2%^(49)^	35(71.4%)	2(4%)	7(14.2%)	5(10.2%)
1-2 years 11.3%^(22)^	14(63.6%)	2(9%)	4(18%)	2(9%)
2-3 years 26.2%^(51)^	18(35.2%)	5(9.8%)	20(39.2%)	8(15.6%)
3-4 years 20.1%^(39)^	2(2.5%)	4(10.2% )	20(51.2%)	13(33.3%)
4-5 years 17.1%^(33)^	2(6%)	8(24%)	15(45.4%)	8(24.4%)

Around 73% of patients with large perforation had moderate mixed hearing loss, while 62.9% had mild mixed hearing loss (Table.3).

**Table 3:** Correlation of the perforation size and hearing loss

**Perforation 115**	**Mild CHL31 (26.9%)**	**Moderate CHL31** **(26.9%)**	**Mild mixed HL** **27 (23.4%)**	**Moderate mixed HL26 (22.6%)**
Small 35 (30.0%)	27(87.09%)	5(16.1%)	2(7.4%)	1(3.8%)
Medium 40 (35.0%)	3 (8.8%)	23 (83.8%)	8 (29.6%)	6 (23%)
Large 40 (35.0%)	1 (3.2%)	3 (9.6%)	17 (62.9%)	19 (73%)

HL: Hearing loss, CHL: Conductive *hearing* loss

Patients with marginal perforation had a higher occurrence of mixed hearing loss. Around 36.8% had moderate mixed hearing loss, while 42.1% had mild mixed hearing loss (Table.4).

**Table 4:** Hearing loss in patients with chronic otitis media squamosal

	Mild CHL	Moderate CHL	Mild mixed HL	Moderate mixed HL
Squamosal disease 79	18 (22.7%)	23 (29.1%)	19 (24.0%)	19 (24.0%)
Attic retraction 16 (20%)	4 (22.2%)	5 (21.7%)	4 (21.0%)	3 (15.7%)
Attic perforation 18 (22.7%)	3 (16.6%)	5 (21.7%)	4 (21.0%)	6 (31.5%)
Marginal perforation 28 (35.4%)	4 (22.2%)	9 (39.1%)	8 (42.1%)	7 (36.84%)
Posterior superior retraction pocket 17 (21.5%)	7 (38.8%)	4 (17.39%)	3 (15.7%)	3 (15.7%)

HL: Hearing loss, CHL: Conductive *hearing* loss

*Cochlear* *involvement* *in chronic otitis media patients* *with* *mixed* *hearing* *loss*

Out of the 194 ears included in the study, 102 had mixed hearing loss. About 45.0% (n=46) had mild mixed hearing loss and of these 93.4% (n=43) were SISI positive. Moderate mixed hearing loss was observed in 54.9% (n=56) ears, of which 94.6% (n=53) were SISI positive. Of the patients with mixed hearing loss, 94.1% (n=96) were SISI positive.

*Cochlear* *involvement* *in* *the control* *group *

In the control group, the SNHL was observed in 5.1% (n=10) cases. Of these individuals, 40% (n=4) had cochlear involvement.

*Comparison* *of the cochlear* *involvement* *in chronic otitis media patients* *and the control group*

Mixed hearing loss was observed in 52.5% (n=102) ears of COM, of which 94.1% (n=96) had positive SISI. The SNHL was noted in 5.1% (n=10) ears in the control group, of which 40% (n=4) had positive SISI. 

## Discussion

The CSOM is a common condition in ENT practice. Conventionally, the CSOM has been associated with the conductive component of hearing loss. The current study was conducted to evaluate the presence of a sensorineural element in hearing loss associated with CSOM. The present study was conducted on 237 subjects with the age range of 11-45 years, consisting of 148 (56%) males and 126 (46%) females. The occurrence of SNHL in CSOM is a well-established fact (2,3). In our study, SNHL was defined as a loss in bone conduction of more than 20 dB at any one or more of the frequencies between 250 to 4,000 HZ. The incidence was found to be estimated at 52%. The effect of COM on bone conduction thresholds is caused as a result of damage to the cochlea or due to alteration in the sound transmission mechanism of the ear. The permeability of the round window membrane increases due to chronic middle ear inflammation. As a result, microtoxins diffuse into the inner ear and damage the inner ear structures. The atrophic changes that normally occur in the supporting tissue of the cochlear duct accelerate. This changes the mass, stiffness, and friction of the spiral ligament or of the basilar membrane, which in turn affects the movement of the cochlear partition in response to sound, thereby leading to SNHL for middle and high frequencies (5-8).The occurrence of SNHL in CSOM as reported by various studies (including the present study) is shown in (Table.5).

**Table 5:** Occurrence of the sensorineural hearing loss in chronic suppurative otitis media patients

**Study was conducted by**	**SNHL cases (%)**
Paperella et al. (8) (1984)	43%
Kaur K et al. (7) (2003)	24%
Sharma K et al. (9) (2005)	9.4%
de Azevedo AF et al. (6) (2007)	13%
Present study (2014)	69.2%

SNHL: Sensorineural hearing loss

Previous studies have suggested that patients with a longer duration of the disease were more likely to experience SNHL (6,7,10-12). Moreover, treatment with eardrops containing ototoxic agents has been implicated in the causation of SNHL [1]. The literature review has shown that greater perforation sizes increase the chance of developing SNHL (9,10).

Based on the findings of the present study, the occurrence of SNHL in CSOM was highest in the age group of 40-45 years (69.2%). In order to rule out the possible effects of presbycusis, patients with the age > 45 years were excluded. Longer duration of ear discharge resulted in a steady increase in the SNHL incidence. Around 71.4% of patients with ear discharge for more than 5 years developed SNHL.

In addition, no patient with ear discharge of less than 1 year developed SNHL. There was no significant correlation between the development of SNHL and the use of topical ototoxic ear drops. 

Patients with pars flaccida perforation were found to have higher chances of developing SNHL (55.2%) than patients with pars tensa perforation (19.7%). It was found that 52% of the patients, who had the mucosal type of disease, had developed SNHL and it was more frequently observed in patients with sub-total and total perforations. In addition, with an increase in the perforation size, the susceptibility to develop SNHL steady increases. 

It can be concluded from the present study that with an increase in the duration of the disease, there is a prolonged exposure of the inner ear to toxins that diffuses through the round window membrane; therefore, resulting in the development of the SNHL. Patients with mucoid ear discharge develop CHL probably due to a lesser amount of microtoxins. 

However, there was no statistically significant difference in the susceptibility to develop CHL or SNHL in patients with mucopurulent or purulent ear discharge. 

Pars flaccida perforation was more frequently associated with cholesteatoma and active middle ear disease was associated with low pH around the round window membrane which facilitates the passage of microtoxins into the inner ear. As the size of perforation increases, more amounts of microtoxins penetrate the inner ear damaging the hair cells, especially at the basal turn of the cochlea which leads to developing SNHL.

## Conclusion

According to the findings of the present research, patients with a longer duration of disease were more likely to develop SNHL. In the case of the mucosal disease, patients with larger perforation size suffered from more severe SNHL. In addition, the occurrence of the SNHL was observed to be higher in patients with the squamosal disease. This could be attributed to the prolonged exposure of the inner ear to toxins that enter via diffusion through the round window membrane. 

These microtoxins damage the hair cells, particularly the basal turn of the cochlea, resulting in the development of the SNHL. Therefore, it is suggested that patients should be counseled early early regarding the need for surgery to prevent from developing irreversible hearing loss. Furthermore, eradication of the disease from the middle ear and early reconstruction of the hearing mechanism during the course of the disease would reduce the burden of SNHL.

Longer duration of middle ear disease results in a higher risk of developing SNHL. The SNHL occurrence is higher in the squamosal and mucosal diseases with larger perforations. Early eradication of the middle ear disease by surgical intervention can prevent irreversible hearing loss.
